# The association between pulmonary embolism and the cancer-related genomic alterations in patients with NSCLC

**DOI:** 10.1186/s12931-020-01437-6

**Published:** 2020-07-16

**Authors:** Wei Xiong, He Du, Wei Ding, Jinyuan Sun, Mei Xu, Xuejun Guo

**Affiliations:** 1 Department of Respiratory Medicine, Xinhua Hospital Affiliated to Shanghai Jiaotong University School of Medicine, No. 1665, Kongjiang Road, Shanghai, 200092 Yangpu District China; 2 Department of Oncology, Shanghai Pulmonary Hospital Affiliated to Tongji University School of Medicine, Shanghai, China; 3 Department of Pulmonary and Critical Care Medicine, Punan Hospital, Pudong New District, Shanghai, China; 4 Department of General Practice, North Bund Community Health Center, Shanghai, Hongkou District China

**Keywords:** NSCLC, Pulmonary embolism, Genomic alterations, Association

## Abstract

To date, the association between the acute pulmonary embolism (PE) and the currently existing cancer-related genomic alterations in patients with non-small cell lung cancer (NSCLC) has been understudied. We reviewed patients with a confirmed histopathological diagnosis of NSCLC who underwent computed tomography pulmonary angiography (CTPA) and molecular tests including ALK, ROS1, EGFR, BRAF V600E as well as PD-L1 during the diagnosis of NSCLC, to explore the association between the genomic alterations and PE. The results showed that, for the patients with positive results of genomic alterations, the proportion of positive ALK (13.6%vs8.5%, *P*<0.001) and PD-L1 (24.7%vs19.9%, *P* = 0.001) in PE group were more than those in Non-PE group. The patients with positive ALK and PD-L1 had the most (19.0%) and second most (15.4%) incidence of PE among all the patients being studied. A multivariate Logistic regression analysis showed that the positive ALK [1.685(1.065–2.215)(*P*<0.001)] and PD-L1[1.798(1.137–2.201)(*P*<0.001)] were correlated with the occurrence of PE. The positive results of ALK and PD-L1 genomic alterations may indicate an increased risk of pulmonary embolism in patients with NSCLC.

## Introduction

Lung cancer has high morbidity and mortality [1]. It is one of many cancers which carry the highest risk of acute pulmonary embolism (PE). PE that is one of the subtypes of venous thromboembolism (VTE), is the third acute cardiovascular syndrome behind myocardial infarction and stroke in the world [2, 3].

A series of previous studies suggested that the risk of PE occurrence was prevalently high in patients with lung cancer especially non-small cell lung cancer (NSCLC) [4–7]. NSCLC especially adenocarcinoma were considered to be the independent risk factors for the occurrence of PE [4, 7]. For NSCLC patients who are concomitant with thrombosis, their survival rate was lower than those without thrombosis [5].

In the advanced NSCLC, molecular tests are frequently used for the detection of cancer-related genomic alterations to identify whether or not targeted therapies or immunotherapy are applicable. The predictive genomic alterations for NSCLC mainly include the anaplastic lymphoma kinase (ALK) fusion oncogene, the C-ros oncogene 1 receptor tyrosine kinase (ROS1) gene rearrangements, the sensitizing epidermal growth factor receptor (EGFR) gene mutations, the B-rapidly accelerated fibrosarcoma (BRAF) V600E point mutations, and the programmed cell death 1-ligand 1 (PD-L1) expression [8]. To date, the association between the aforementioned cancer-related genomic alterations and PE in patients with NSCLC has been understudied yet. Therefore the present study was aimed at this subject.

## Methods

### Study design

A retrospective study was performed to investigate the association between the acute pulmonary embolism and the currently existing cancer-related genomic alterations in patients with NSCLC. All the patients with NSCLC of three hospitals in Shanghai between Jan, 2017 and Dec, 2019 were reviewed. The results of cancer-related genomic alterations in the molecular tests of ALK, ROS1, EGFR, BRAF V600E, and PD-L1 were compared between NSCLC patients with PE who were defined as PE group and those without PE who were defined as Non-PE group. The proportion of patients with PE was compared among the patients with positive results of ALK, ROS1, EGFR, BRAF V600E, as well as PD-L1 and those with negative results of molecular tests. The association between PE and the results of cancer-related genomic alterations was analyzed. The data of patients being studied were retrieved from the electronic medical record system (EMRS) of those three hospitals in Shanghai. This protocol was approved by Shanghai Xinhua Hospitals, Shanghai Pulmonary Hospital and Shanghai Punan Hospital.

### Study population

The inclusion criteria comprised: 1) all eligible patients were older than 18 years; 2) all eligible patients had a confirmed histopathological diagnosis of NSCLC; 3) all eligible patients underwent a computed tomography pulmonary angiography (CTPA) which could confirm the presence or absence of PE during the diagnosis of NSCLC; The indications for the CTPA examination included: patients had a moderate or high clinical pretest probability(C-PTP) of PE which was generally assessed by using Wells score [9] or Geneva score [10] and/or an elevated D-dimer level higher than 500 ng/ml [11]; the structure of lumps, nodules and pulmonary vasculature in lungs required to be investigated before the bronchoscopic lung biopsy, percutaneous lung biopsy and pneumonectomy; 4) all eligible patients took the molecular tests of ALK, ROS1, EGFR, BRAF V600E and PD-L1 during the diagnosis of NSCLC. The molecular tests were performed routinely during the diagnosis of NSCLC regardless of the tumor staging in the participating hospitals. The histopathological specimen used for the molecular tests were taken from the pathological tissue obtained during the diagnosis of NSCLC. The exclusion criteria comprised: 1) patients had other known cancers apart from NSCLC; 2) patients had a history of chronic pulmonary embolism.

### Measurements

For all eligible patients being included into the final analysis, the following measurements were performed. Firstly, between PE group and Non-PE group, the overall positive incidence of molecular testing was compared. Meanwhile, the respective positive incidence of ALK, ROS1, EGFR, BRAF V600E and PD-L1 (≥1%) [8] were also compared between two groups. Secondly, for NSCLC patients with different results of molecular tests, the proportion of patients with PE were compared among patients with positive ALK, ROS1, EGFR, BRAF V600E, as well as PD-L1 and the negative results of molecular tests. Thirdly, the association of molecular tests including ALK, ROS1, EGFR, BRAF V600E, as well as PD-L1and the incidence of PE was analyzed.

### Statistical analyses

Measurement data were presented as mean ± standard deviation or median with interquartile range according to whether or not they conformed to normal distribution. Categorical data were presented as frequencies and percentages. Comparison of measurement data between or among groups was performed by using T-test or ANOVA. Comparison of rates among groups was performed by Chi-square test. The association between the genomic alterations of ALK, ROS1, EGFR, BRAF V600E as well as PD-L1 and the incidence of PE was analyzed by using univariate and multivariate Logistic regression analysis. A *P*-value being less than 0.05 was defined as statistical significance.

## Results

### The demographics and clinical characteristics of patients

A total of 1320 patients were included into the study by the inclusion criteria. After the exclusion of 108 patients with other known cancers apart from NSCLC and 25 patients with a history of chronic pulmonary embolism, 1187 patients entered the final analysis. The mean age of all subjects was 66.5 years old. The number of female and male patients were 571 and 616, respectively. Among all 1187 patients, the number of the patients with PE and those without PE were 141 and 1046, respectively, while the number of the patients with positive molecular results and those with negative results were 633 and 554, respectively. For 141 patients with PE, 81 had positive molecular results whereas 60 had negative ones. For 1046 patients without PE, 552 had positive molecular results whereas 494 had negative ones. The number of PE and Non-PE in patients with positive ALK, ROS1, EGFR, BRAF V600E and PD-L1 were 11/47, 5/37, 42/335, 3/23, and 20/110, respectively. The demographics and clinical characteristics of patients being studied are summarized in Table 1.

**Table 1:** The Demographics and clinical characteristics of the patients being studied

	PE(*n* = 141)	Non-PE(*n* = 1046)	*P* value
Age-years	68.2 (64.9–71.5)	64.8 (56.5–73.1)	0.814
Sex-female/male-no.	68/73	503/543	0.975
Smoking -Y/N-no.	81/60	418/628	<0.001
Smoking index -pack-year	47.5 (36.2–56.4)	31.8 (22.2–40.1)	0.025
Histopathology-no. (%)			
Adenocarcinoma	125 (88.7)	938 (89.7)	0.279
Squamous	12 (8.5)	80 (7.6)	0.719
Other	4 (2.8)	28 (2.7)	0.912
Stage-no. (%)			
Stage I	5 (3.5)	231 (22.1)	<0.001
Stage II	26 (18.4)	260 (24.9)	0.094
Stage III	42 (29.8)	303 (29.0)	0.841
Stage IV	68 (48.3)	252 (24.0)	<0.001
C-PTP of PE- no. (%)			
Low	21 (14.9)	632 (60.4)	<0.001
Moderate	45 (31.9)	253 (24.2)	0.047
High	75 (53.2)	94 (15.4)	<0.001
Concomitant DVT- no. (%)	45 (31.9)	52 (5.0)	<0.001
D-dimer- ng/ml	4680 (2440–6920)	808 (678–938)	<0.001
Molecular tests-P/N-no.	81/60	552/494	0.296
ALK (+) - no. (%)	11 (13.6%)	47 (8.5%)	<0.001
ROS-1(+) - no. (%)	5 (6.2%)	37 (6.7%)	0.858
EGFR (+) - no. (%)	42 (51.8%)	335 (60.7%)	0.130
BRAF- V600E (+) - no. (%)	3 (3.7%)	23 (4.2%)	0.845
PDL-1(+) (≥1%) - no. (%)	20 (24.7%)	110 (19.9%)	0.001

Note: *PE* Pulmonary embolism, *no.* Number, *Y* Yes, *N* No, *C-PTP* Clinical pretest probability, *DVT* Deep venous thromboembolism, *ng* Nanogram, *ml* Milliliter, *P* Positive, *N* Negative, *ALK* Anaplastic Lymphoma Kinase, *ROS1* C-Ros Oncogene 1 Receptor Tyrosine Kinase, *EGFR* Epidermal Growth Factor Receptor, *BRAF* B-Rapidly Accelerated Fibrosarcoma, *PD-L1* Programmed Cell Death 1-Ligand 1

### The comparison of positivity of molecular testing between PE and non-PE group

Between PE group and Non-PE group, no statistical difference was found with respect to the overall incidence of positive molecular tests (57.4% vs 52.8%, *P* = 0.296). Nevertheless, with respect to each of ALK, ROS1, EGFR, BRAF V600E and PD-L1, the results showed that, the proportion of positive ALK (13.6%vs8.5%, *P*<0.001) and PD-L1 (24.7%vs19.9%, *P* = 0.001) in PE group were more than those in Non-PE group, whereas no statistical difference was found with respect to ROS1, EGFR, and BRAF V600E between PE and Non-PE group. (Table 1.)

### The comparison of PE incidence among the NSCLC patients with different results of molecular tests

**Fig. 1:**
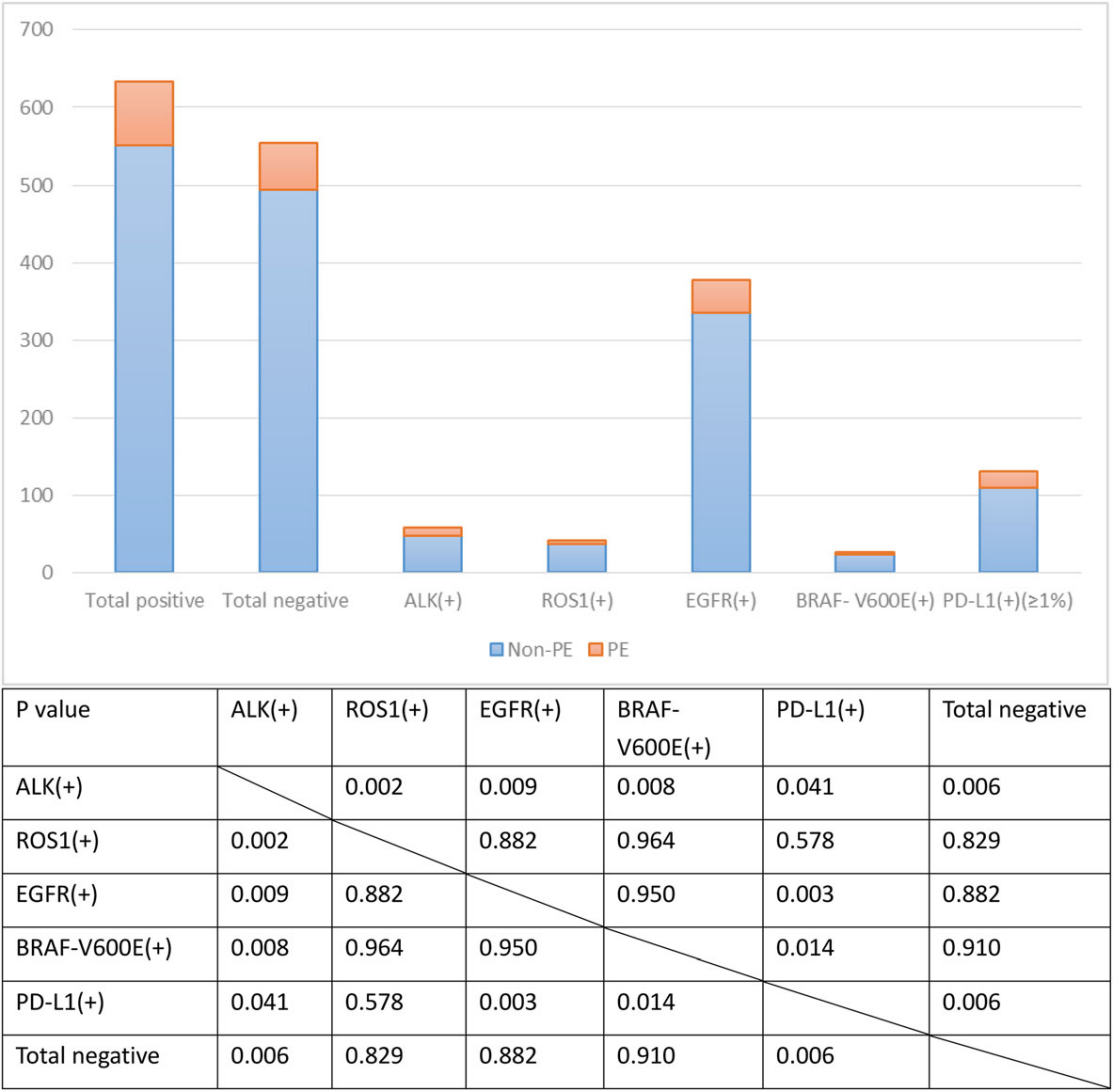
The comparison of PE incidence among the NSCLC patients with different results of molecular tests. Note: ALK: Anaplastic Lymphoma Kinase; ROS1: C-Ros Oncogene 1 Receptor Tyrosine Kinase; EGFR: Epidermal Growth Factor Receptor; BRAF: B-Rapidly Accelerated Fibrosarcoma; PD-L1: Programmed Cell Death 1-Ligand 1

The proportion of patients with PE between the patients with positive and negative molecular tests was 81/633(12.8%) versus 60/554(10.8%). (*P* = 0.296). The proportion of patients with PE among the patients with positive ALK, ROS1, EGFR, BRAF V600E and PD-L1 were 19.0, 11.9, 11.1, 11.5, and 15.4%, respectively. In comparison, the patients with positive ALK and PD-L1 had the most and second most incidence of PE among all the patients being studied. The diagram of comparison of PE incidence among the NSCLC patients with different results of molecular tests is illustrated in Fig. 1.

### The correlation between the results of molecular tests and PE in patients with NSCLC

An univariate Logistic regression analysis showed that the positive molecular results [1.265(0.9141.537)(*P* = 0.025)], ALK(+)[1.576(1.0282.126)(*P* = 0.001)],ROS1(+)[1.337(0.8781.990)(*P* = 0.030)], EGFR(+)[0.838(0.596–1.157) (*P* = 0.025)], BRAF-V600E(+)[0.987(0.774–1.811)(*P* = 0.032)], and PD-L1(+) [1.437(0.975–2.018)(*P* = 0.005)],were correlated with the occurrence of PE in the patients being studied. The following multivariate Logistic regression analysis showed that the positive ALK [1.685(1.065–2.215)(*P*<0.001)] and PD-L1[1.798(1.137–2.201)(*P*<0.001)] remained to be correlated with the occurrence of PE, whereas no significant correlation was found between them with regard to ROS1, EGFR, and BRAF V600E.(Table 2.)

**Table 2:** The correlation between the results of molecular tests and PE in patients with NSCLC

	Odds ratio (Univariate)	Odds ratio (Multivariate)
Age (per increase of ten years)	1.257 (0.519–1.595)(*P* = 0.020)	1.232 (0.661–1.822)(*P* = 0.100)
Sex (male as 1.0)	1.160 (0.487–1.673)(*P* = 0.098)	
Smoking (non-smoker as 1.0)	1.396 (0.812–1.934)(*P* = 0.010)	1.518 (1.054–2.099)(*P* = 0.001)
D-dimer (per increase of 500 ng/ml)	1.779 (1.284–2.318) (*P* = 0.001)	1.762 (1.236–2.243) (*P*<0.001)
Concomitant DVT (no DVT as 1.0)	2.346 (1.832–2.818)(*P*<0.001)	2.859 (1.971–2.945)(*P*<0.001)
Positive molecular results (negative results as 1.0)	1.265 (0.914–1.537)(*P* = 0.025)	1.187 (0.674–1.532) (*P* = 0.176)
ALK(+)(negative results as 1.0)	1.576 (1.028–2.126)(*P* = 0.001)	1.685 (1.065–2.215)(*P*<0.001)
ROS1(+) (negative results as 1.0)	1.337 (0.878–1.990)((*P* = 0.030)	1.293 (0.921–2.006)((*P* = 0.125)
EGFR(+) (negative results as 1.0)	0.838 (0.596–1.157)((*P* = 0.025)	0.907 (0.614–1.289)((*P* = 0.352)
BRAF-V600E(+) (negative results as 1.0)	0.987 (0.774–1.811)(*P* = 0.032)	1.050 (0.567–1.935)(*P* = 0.212)
PD-L1(+)(negative results as 1.0)	1.437 (0.975–2.018)(*P* = 0.005)	1.798 (1.137–2.201)(*P*<0.001)
Platelet (per increase of 100 × 10^9^ /L)	1.654 (1.125–2.187)(*P* = 0.001)	1.731 (1.292–2.318) (*P*<0.001)

Note: *PE* Pulmonary embolism, *NSCLC* Non-small cell lung cancer, *ng* Nanogram, *ml* Milliliter, *DVT* Deep venous thromboembolism, *ALK* Anaplastic Lymphoma Kinase, *ROS1* C-Ros Oncogene 1 Receptor Tyrosine Kinase, *EGFR* Epidermal Growth Factor Receptor, *BRAF* B-Rapidly Accelerated Fibrosarcoma, *PD-L1* Programmed Cell Death 1-Ligand 1

## Discussion

In the current study, it was discovered that the positive ALK and PD-L1 may indicate an increased risk of pulmonary embolism in patients with NSCLC. The reason why we chose the newly-diagnosed patients with NSCLC was that the newly-diagnosed patients may reflect the natural relationship between PE and the results of molecular tests in patients with NSCLC, since the incidence of PE will be interfered with surgery, chemotherapy and so on, along with the initiation of the oncotherapy [12].

With respect to the results of a handful of comparable studies, part results of the current study are basically consistent with the most of them. In the study of Lee et al., no relation was revealed between EGFR mutation or ALK rearrangements and the VTE risk of Asian patients with NSCLC. However, the correlation was assessed between the molecular mutation and clinically diagnosed PE without the confirmation via CTPA in the study of Lee et al. [13] In the study of Corrales-Rodriguez et al. [14] and of Verso et al. [15], EGFR was not associated with an increased risk of VTE in patients with NSCLC, being consistent with the current study. In the study of Zer et al., the rate of VTE in an ALK-rearranged cohort was 3 to 5 fold higher than previously reported in NSCLC population [16]. In the study of Dou et al., the presence of ALK rearrangement was associated with increased risk of VTE in patients with NSCLC [17]. In a most recent study, the time-to-event analyses indicated that the ALK rearrangement resulted in a 4-fold increase of VTE risk and 3-fold increase of arterial thrombosis risk in NSCLC [18]. These findings are consistent with our findings. To date, the possible reason why ALK causes VTE may be due to that the ALK rearrangement may increase the risk of VTE by enhancing tissue factor procoagulant activity via NF-κB signaling pathway, or by mucin abundance, or by heparin [16, 17].

With regard to ROS-1, being inconsistent with our finding, a recent study reported that the incidence of VTE which included 46.4% of PE, was 3 to 5 fold higher in patients with ROS1-rearrangment than that in general population with NSCLC [19]. Nevertheless, the study merely measured 48 patients, making large-scale investigation necessary to confirm the conclusion. For BRAF and PD-L1, there have been no comparable studies. The underlying mechanism of the high incidence of pulmonary embolism in PD-L1-positive patients is still unclear. A previous study demonstrated that there was a positive correlation between sPD-1 levels and platelet counts [20]. As a result, thrombocytosis in PD-L1-positive patients may be the predisposing factor of pulmonary embolism. It was also observed that the risk of having a PE increased along with the increase of platelets in the present study. Nevertheless, this is merely a hypothesis that needs to be further verified.

Some clinical implications may derive from the current study. On account of the current findings, clinicians are suggested to be vigilant and thinking of the higher probability of PE incidence in NSCLC patients with positive ALK and PD-L1, in comparison to those without them. Accordingly, the clinicians’ awareness of screening, prophylaxis, and follow-up for underlying PE should be enhanced for this patient population. As a result, the monitoring of C-PTP of PE and D-dimer level should be more frequently performed to rule out the underlying PE, for NSCLC patients with positive ALK and PD-L1, in comparison with the opposite. Meanwhile, the implementation of prophylactic anticoagulation should be taken into account more often in NSCLC patients with positive ALK and PD-L1 than in those without them, especially when there is a moderate or high C-PTP of PE and/or an elevated D-dimer level.

This study suffers from some limitations. First of all, this is a retrospective study which may have certain bias. A prospective study is warranted in the future. Secondly, the exclusion of PE was implemented by using CTPA in the current study, however, some most distally peripheral sub-segmental embolism may not be detected on CTPA. The combined examination of CTPA and pulmonary ventilation perfusion scan may be applicable if a prospective study is carried out.

## Conclusion

In conclusion, the current study suggests that the positive results of ALK and PD-L1 among existing genomic alterations of NSCLC may indicate an increased risk of pulmonary embolism in patients with NSCLC. The message being delivered hereby may be conducive to the screening and prophylaxis of pulmonary embolism in patients with NSCLC in daily clinical practice.

## Data Availability

The datasets used and/or analysed during the current study are available from the corresponding author on reasonable request.

## Abbreviations

NSCLC: Non-Small Cell Lung Cancer
PE: Pulmonary Embolism
ALK: Anaplastic Lymphoma Kinase
ROS1: C-Ros Oncogene 1 Receptor Tyrosine Kinase
EGFR: Epidermal Growth Factor Receptor
BRAF: B-Rapidly Accelerated Fibrosarcoma
PD-L1: Programmed Cell Death 1-Ligand 1
CTPA: Computed Tomography Pulmonary Angiography
VTE: Venous Thromboembolism
DVT: Deep Venous Thromboembolism
